# Immune checkpoint inhibitor therapy and elevated levels of C-reactive protein associated with COVID-19 aggravation in patients with lung cancer

**DOI:** 10.1186/s40780-022-00259-6

**Published:** 2022-11-01

**Authors:** Masatoshi Maki, Ryo Takada, Akihiko Taniguchi, Naoyuki Nomura, Seiichiro Kuramoto, Yuki Chiko, Toshiaki Okada, Seiji Saito, Koji Tamura

**Affiliations:** 1 Department of Hospital Pharmacy, National Hospital Organization Fukuyama Medical Center, 4-14-17 Okinogami-cho, Fukuyama, Hiroshima 720-8520 Japan; 2 Department of Respiratory Medicine, National Hospital Organization Fukuyama Medical Center, Fukuyama, Hiroshima, Japan; 3 Department of Infection Control, National Hospital Organization Fukuyama Medical Center, Fukuyama, Hiroshima, Japan

**Keywords:** COVID 2019, Immune checkpoint inhibitor, Lung cancer, Respiratory failure, C-reactive protein

## Abstract

**Background:**

COVID-19 has become a significant health threat and a primary healthcare concern among the most vulnerable patients with cancer. Patients with COVID-19 who have lung cancer are at great risk and need careful monitoring if they are affected. This study aimed to investigate the clinical characteristics of COVID-19-positive patients with lung cancer and the risks associated with anticancer medication.

**Methods:**

This study was a single-center, retrospective cohort study. Patients with lung cancer who presented with COVID-19 during hospitalization were divided into two groups: those who presented with respiratory failure and those who did not. The patient's background, clinical laboratory values, and anticancer drugs used for therapy were investigated to identify risk factors for respiratory failure.

**Results:**

Thirty-one patients were included in the study; 18 (58.1%) were in the respiratory failure group and 13 (41.9%) were in the group without respiratory failure. In the respiratory failure group, there was a significant difference in using immune checkpoint inhibitor (ICI) use within 90 days (*p* = 0.025) and the level of C-reactive protein (CRP) level (*p* = 0.017). The analysis of the operating characteristic of the receiver revealed a cutoff value of 2.75 mg/dL for CRP (area under the curve = 0.744, sensitivity 0.611, specificity 0.923).

**Conclusions:**

A history of ICI within 90 days and elevated CRP (≥ 2.75 mg/dL) levels are potential factors leading to respiratory failure in COVID-19-affected patients undergoing chemotherapy for lung cancer.

## Background

Severe Acute Respiratory Syndrome Coronavirus (SARS-CoV-2) has remained globally endemic since the first case in Wuhan, China, was reported in December 2019 [1]. Following an initial outbreak in January 2020, the episode in Japan has been repeatedly contained and re-emerged, with clusters occurring in medical facilities and nursing homes, killing many infected patients.

The severity of Coronavirus disease 2019 (COVID-19) includes malignancy, chronic lung disease, and older adults [2–4]. Patients with cancer are vulnerable to infections due to chronic illnesses and poor health conditions [5, 6]. Furthermore, the immunosuppression caused by cancer and anticancer drug therapy causes the patients to be more susceptible to the severity of the disease [7, 8]. Previous research has also suggested that patients with cancer are more vulnerable to SARS-CoV-2 than patients without cancer [6]. Thus, COVID-19 has been associated with a higher risk of severeness in patients with lung cancer [7, 9]. Furthermore, given the impact of lung cancer treatment, the Thoracic Cancers International COVID-19 Collaboration has shown that the use of cytotoxic anticancer drugs for three months has increased COVID-19-related mortality [10]. Moreover, immune checkpoint inhibitors (ICI) have contributed to the severity of the COVID-19 illness in patients with cancer [11].

In contrast, some studies have found that cytotoxic anticancer drugs do not affect COVID-19-related mortality and suggest that ICIs do not affect the severity of the COVID-19 illness [12, 13]. Therefore, the risk factors associated with the severity of COVID-19 in patients with lung cancer undergoing chemotherapy are unknown. To the best of our knowledge, no studies have been conducted in the Japanese population to investigate the risk factors in COVID-19-positive patients with a history of lung cancer.

In this study, we retrospectively examined risk factors for the severity of COVID-19 in patients with lung cancer who were diagnosed with COVID-19 during hospitalization and who had developed or did not develop respiratory failure during the observation period. This study aimed to identify risk factors for severe disease in patients with lung cancer and to identify cases that require careful monitoring.

## Methods

### Study design and participants

A single-center, retrospective cohort study was conducted. The study included adult patients with positive SARS-CoV-2 reverse transcription-polymerase chain reaction tests who received chemotherapy for advanced or recurrent lung cancer or postoperative adjuvant chemotherapy for lung cancer at Fukuyama Medical Center in Hiroshima, Japan, between December 1, 2020, and February 28, 2021.

**Fig. 1:**
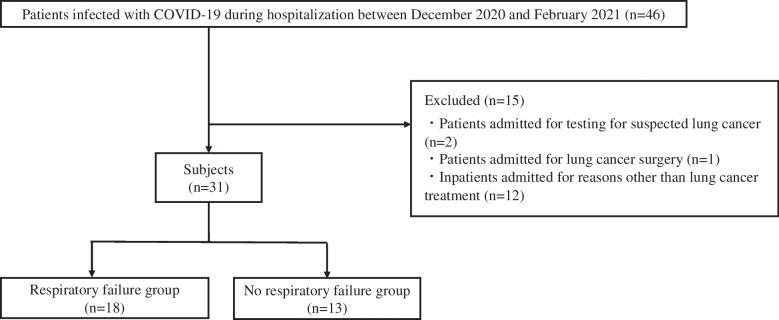
Flowchart of patient selection

The following patients were excluded: those admitted for tests for suspected lung cancer, those scheduled for lung cancer surgery, and those admitted for treatments other than lung cancer (Fig. 1).

Details of age, sex, smoking history, comorbidities [interstitial pneumonia, chronic obstructive pulmonary disease, diabetes mellitus, chronic kidney disease, hypertension, obesity (body mass index ≥ 30)], presence or absence of pre-onset oxygen (O_2_), additional antiviral medications, anticoagulation therapy, outcome (death or improvement), blood biochemistry, anticancer drugs used prior to disease onset, lung cancer histology, and treatment line were obtained from patient’s files.

For laboratory values, we used blood test data collected within three days of the SARS-CoV-2 detection. We investigated whether or not a cytotoxic anticancer drug was administered within 14 days of the onset of anticancer medications before the start of the disease. Based on a previous report that highlighted the possibility of varying severity of COVID-19 depending on the total number of ICI doses and the time between the last ICI dose and the diagnosis of COVID-19 [14], this study investigated the prognostic impact of the total number of ICI doses and the time between the last ICI dose and the onset of COVID-19 in patients who had previously received ICIs, within 90 days or not.

### Clinical outcomes

Aggravation was defined as the death or development of respiratory failure within 30 days of a positive nucleic acid detection test. Factors associated with exacerbations were also examined.

### Study subgroups

The patients were classified into the respiratory failure group or the group without respiratory failure, and their backgrounds and laboratory values were compared and examined. Respiratory failure was defined in the simplest way possible manner to ensure feasibility. The groups were defined as follows:

- Respiratory failure group: percutaneous arterial blood O_2_ saturation (SpO_2_) ≤ 93%, with O_2_ administration during the disease course. This corresponds to a score ≥ 5 on the WHO clinical progression scale for COVID-19, as proposed by the WHO Working Group [15].
- Group without respiratory failure: SpO_2_ > 93%, without O_2_ administration during the disease course. This corresponds to a score ≤ 4 on the WHO clinical progression scale for COVID-19, as proposed by the WHO Working Group [15].

### Statistical analysis

Data are expressed as median (range). The Mann − Whitney U test was used to analyze continuous variables, Fisher's exact test was used to compare nominal measures, and the Bonferroni method was used to test between multiple groups. For items with significant continuous variables, cutoff values were obtained from receiver operating characteristic (ROC) analysis. In the univariate analysis, the Kaplan − Meier method was used to calculate the time until respiratory failure for significant items. The log-rank test was used for comparisons between groups. Two-sided *p* < 0.05 was considered statistically significant. All statistical analyses were performed using EZR software (ver 1.54) (Saitama Medical Center, Jichi Medical University, Saitama, Japan).

### Ethical matters

This study was conducted in compliance with the “Ethical Guidelines for Medical Research Involving Human Subjects” and the “Appropriate Handling of Personal Information by Medical and Nursing Care Providers” guidelines and was approved by the Ethics Review Committee of the hospital (Fukuyama Medical Center Ethics Review Committee, Approval number: R3-13).

## Results

### Patient background

**Fig. 2:**
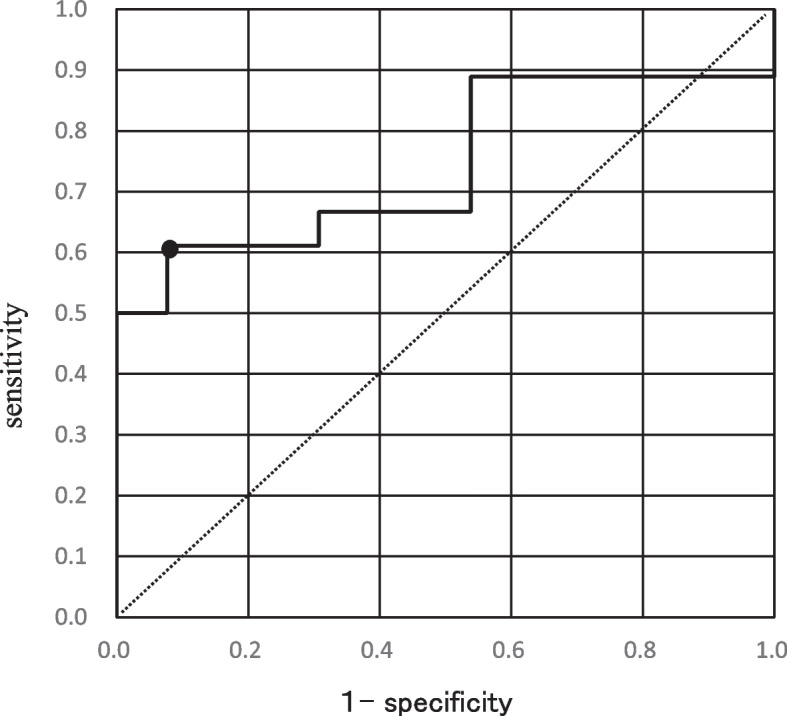
Receiver operating characteristic (ROC) analysis of C-reactive protein (CRP) level for respiratory failure. The cutoff value of CRP level for the presence of respiratory failure was determined by the ROC analysis. The cutoff value of CRP level was 2.75 mg/dL, with an area under the curve (AUC) of 0.744, sensitivity of 0.611, and specificity of 0.923

Thirty-one patients were included in the study: 18 (58.1%) and 13 (41.9%) patients with respiratory failure and no respiratory failure groups, respectively. Age, sex, smoking history, presence of comorbidities (interstitial pneumonia, chronic obstructive pulmonary disease, diabetes, chronic kidney disease, hypertension, and obesity), and whether O_2_ was administered before the onset of the disease were not significantly different between the two groups. Furthermore, there were no significant differences between the two groups in the use of cytotoxic anticancer drugs within 14 days. Regarding the history of ICI administration within 90 days, seven patients (38.9%) were in the respiratory failure group, which was significantly higher (*p* = 0.025) than the zero patients (0.0%) in the group without respiratory failure. The ICIs used in this study were pembrolizumab, an anti- programmed cell death 1 antibody, in three patients; and atezolizumab, an anti-programmed cell death ligand 1 antibody, in four patients. No cytotoxic T lymphocyte antigen-4 inhibitors were used. Furthermore, there were no significant differences between the two groups in terms of histologic type of lung cancer or line of treatment. No significant differences were found between the two groups regarding lymphocyte count, platelet count, D-dimer, aspartate aminotransaminase, alanine aminotransferase, serum creatinine, and lactate dehydrogenase; however, C-reactive protein (CRP) was significantly higher in the respiratory failure group (*p* = 0.017) (Table 1). Consequently, a ROC analysis was performed for CRP, as it was the only significant laboratory value. The cutoff value obtained by the ROC analysis for CRP was 2.75 mg/dL (area under the curve, 0.744; sensitivity, 0.611; specificity, 0.923) (Fig. 2).

**Table 1:** Baseline characteristics of the two subgroups

Characteristic	Total (*n* = 31)	Respiratory failure (*n* = 18)	No respiratory failure (*n* = 13)	*p* value
Median age years (range)	72 (4686)	72 (50 − 81)	73 (46 − 86)	0.297^a)^
65 years old and over, n (%)	25 (80.6%)	14 (77.8%)	11 (84.6%)	1.000^b)^
Male, n (%)	27 (87.1%)	17 (94.4%)	10 (76.9%)	0.284^b)^
Smoking history, yes, n (%)	26 (83.9%)	17 (94.4%)	9 (69.2%)	0.134^b)^
Comorbidities, n (%)				
Interstitial pneumonia	10 (32.3%)	7 (38.9%)	3 (23.1%)	0.452^b)^
Chronic obstructive pulmonary disease	18 (58,1%)	12 (66.7%)	6 (46.2%)	0.294^b)^
With pre-onset O_2_ administration	4 (12.9%)	4 (22.2%)	0 (0%)	0.120^b)^
Diabetes	11 (35.5%)	5 (27.8%)	6 (46.2%)	0.449^b)^
Chronic kidney disease	10 (32.3%)	8 (44.4%)	2 (15.4%)	0.129^b)^
Hypertension	11 (35.5%)	9 (50.0%)	2 (15.4%)	0.070^b)^
Obesity (BMI over 30)	2 (6.5%)	1 (5.6%)	1 (7.7%)	1.000^b)^
History of administration of cytotoxic anticancer drugs within 14 days, n (%)	17 (54.8%)	10 (55.6%)	7 (53.8%)	1.000^b)^
History of ICI administration within 90 days, n (%)				
Total	7 (22.6%)	7 (38.9%)	0 (0%)	0.025^b),*^
Atezolizumab	4 (12.9%)	4 (22.2%)	0 (0%)	-
Pembrolizumab	3 (9.7%)	3 (16.7%)	0 (0%)	-
small cell carcinoma	3 (9.7%)	1 (5.6%)	2 (15.4%)	0.198^b)^
squamous cell carcinoma	13 (41.9%)	10 (55.6%)	3 (23.1%)	
adenocarcinoma	14 (45.2%)	7 (38.9%)	7 (53.8%)	
mesothelioma	1 (3.2%)	0 (0%)	1 (7.7%)	
1st line	11 (35.5%)	7 (38.9%)	4 (30.8%)	0.981^b)^
2nd line	6 (19.4%)	4 (22.2%)	2 (15.4%)	
3rd line and above	13 (41.9%)	7 (5.6%)	6 (7.7%)	
adjuvant chemotherapy	1 (3.2%)	0 (0%)	1 (7.7%)	
Laboratory data Median [min–max]				
White blood cell (× 10^3^/µL)		5.0 [1.3 − 11.9]	4.3 [1.6 − 6.9]	0.400^a)^
Neutrophil (× 10^3^/µL)		3.4 [0.4 − 7.9]	2.8 [0.6 − 5.9]	0.489^a)^
Lymphocyte (× 10^3^/µL)		0.8 [0.2 − 3.1]	0.7 [0.5 − 1.3]	0.357^a)^
Hemoglobin (g/dL)		10.7 [7.9 − 13.6]	11.3 [9.4 − 13.9]	0.575^a)^
Platelets (× 10^3^/µL)		193.5 [75 − 424]	165 [27 − 280]	0.562^a)^
Albumin (g/dL)		3.2 [2.2 − 4.0]	3.6 [2.8 − 4.4]	0.296^a)^
LDH (U/L)		234.5 [140 − 418]	206 [139 − 426]	0.483^a)^
AST (U/L)		22 [9 − 44]	28 [17 − 45]	0.054^a)^
ALT (U/L)		19 [6 − 50]	20 [8 − 77]	0.561^a)^
Creatinine (mg/dL)		0.9 [0.49 − 1.54]	0.9 [0.53 − 1.5]	0.305^a)^
CRP (mg/dL)		3.79 [0.04 − 12.6]	1.47 [0.2 − 3.1]	0.017^a),^^*^
D-dimer (µg/mL)		1.3 [0.7 − 10.2]	1.7 [0.7 − 7.9]	0.641^a)^
Ferritin (ng/mL)		419.1 [185.5 − 1251.8]	356.4 [53.2 − 1006.1]	0.481^a)^
KL-6 (U/mL)		543 [176 − 2440]	452 [162 − 1470]	0.474^a)^

*Abbreviations*: *ALT* Alanine aminotransferase, *AST* Aspartate aminotransaminase, *BMI* Body mass index, *CRP*, C-reactive protein, *KL-6* Krebs von den Lungen-6, *LDH* Lactate dehydrogenase

^*^P < 0.05 was considered statistically significant

^a)^ Mann − Whitney U test

^b)^ Fisher's exact test

### Additional or combination medications and mortality

Favipiravir was added to the antiviral regimen in all patients, and treatment started within three days after the diagnosis of COVID-19 diagnosis. Heparin was administered to 11 of the 18 patients (61.1%) and 4 of the 13 patients (30.8%) in the groups with and without respiratory failure, respectively; however, there was no significant difference between the two groups. Steroids were administered to 17 of the 18 patients (94.4%) and 8 of the 13 patients (61.5%) in the groups with and without respiratory failure, respectively; however, there was no significant difference between the two groups. The overall number of deaths during observation was 10 (32%) in the respiratory failure group (Table 2).

**Table 2:** Additional or combination medication and mortality

	Total (*n* = 31)	Respiratory failure (*n* = 18)	No respiratory failure (*n* = 13)	*p* value
Favipiravir, n (%)	31 (100%)	18 (100%)	13 (100%)	1.000^b)^
Heparin, n (%)	15 (48.4%)	11 (61.1%)	4 (30.8%)	0.149^b)^
Steroid, n (%)	25 (80.6%)	17 (94.4%)	8 (61.5%)	0.059 ^b)^
Number of deaths, n (%)	10 (32.2%)	10 (55.6%)	0 (0%)	0.001^b)*^

^*^P < 0.05 was considered statistically significant

^a)^ Mann − Whitney U test

### Examination of factors influencing respiratory failure

Based on the cutoff values obtained in the ROC analysis, the high CRP group was defined as CRP level ≥ 2.75 mg/dL, and the low CRP group as CRP level < 2.75 mg/dL.

**Fig. 3:**
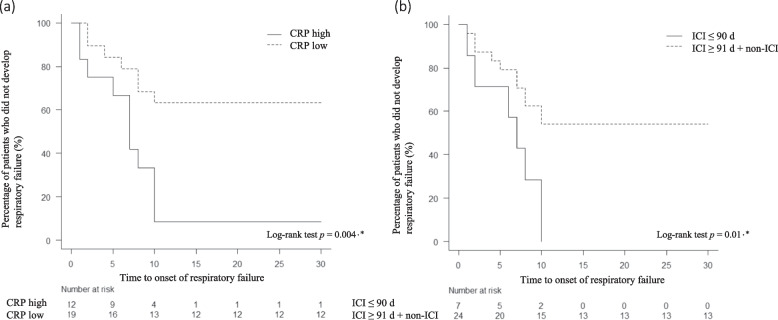
Time to onset of respiratory failure. Time from COVID-19 diagnosis to the onset of respiratory failure. Patients were sub-grouped into (a) high and low C-reactive protein (CRP) groups based on calculated cutoff values as well as (b) groups with and without prior immune checkpoint inhibitor (ICI) administration within 90 days. Log-rank test, *p < 0.05. ICI ≤ 90 d, ICI within 90 days. ICI ≥ 91 d + non-ICI, ICI over 91 days + non-ICI

Since a CRP level above the cutoff value and history of ICI administration within 90 days were significantly associated with respiratory failure in the univariate analysis, we examined the effect of high CRP level and history of ICI administration within 90 days on the time to respiratory failure (Fig. 3).

The high CRP (≥ 2.75 mg/dL) group had a significantly shorter time to respiratory failure than the low CRP (< 2.75 mg/dL) group (*p* = 0.004, Fig. 3a). Patients who had received an ICI within 90 days (“ICI group”) had a significantly shorter time to respiratory failure than those who had not (*p* = 0.01, Fig. 3b). The median time to onset of respiratory failure in the high CRP and ICI groups were 8 (range, 1 − 10) and 8 (range, 1 − 10) days, respectively. If both high CRP level and a history of ICI administration within 90 days were considered risk factors, then there were four patients with both risk factors, all of whom developed respiratory failure. Eleven patients had a high CRP level or a history of ICI within 90 days as risk factors, whereas there were 7 patients who developed respiratory failure and 4 patients who did not develop respiratory failure. There was no statistically significant difference in the development of respiratory failure between patients with two risk factors and those with only one risk factor (*p* = 0.832).

### Prognostic impact of the total number of ICI doses and the time from the last ICI dose to the diagnosis of COVID-19

**Fig. 4:**
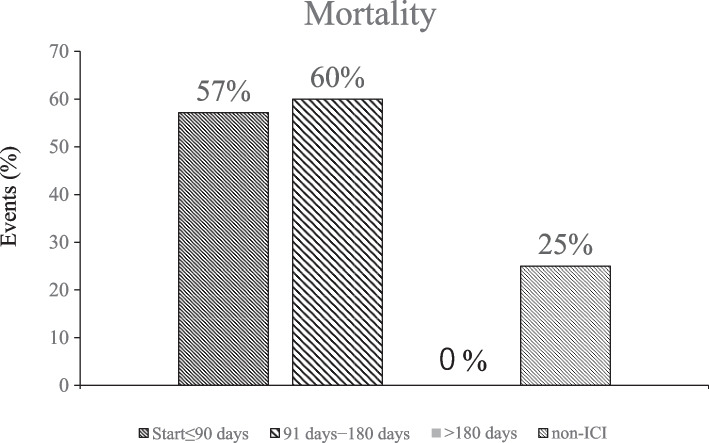
Effect of previous immune checkpoint inhibitor (ICI) administration on COVID-19 prognosis in patients with lung cancer. Patients were categorized into four groups: within 90 days from last ICI administration to COVID-19 diagnosis, 91–180 days, > 180 days, and non-ICI group, and the mortality rate for each group is indicated

The total number of ICI doses, respiratory failure, and mortality in patients with a history of prior ICI administration, whether within 90 days or not, were studied. The median total number of ICI doses was 5.5 (range, 1–18) in the respiratory failure group and 16 (range, 3–32) in the group without respiratory failure; however, this difference between the two groups was not significant (*p* = 0.07). Seven of the 10 patients who died in this study had previously been treated with ICI. In the group with patients that died, the median total number of ICI doses was 4 (range, 1–18), whereas in the survival group, the median total number of ICI doses was 9 (range, 1–32); however, this difference between the two groups was not significant (*p* = 0.27). Additionally, the relationships between the time from the last administration of ICI to the diagnosis of COVID-19 and respiratory failure were examined. The median time from the last administration of ICI to diagnosis was 76 days (range, 10–274 days) in the group with respiratory failure group and 221 days (range, 163–420 days) in the group without respiratory failure group. The time to diagnosis of COVID-19 was significantly shorter in the group with respiratory failure group (*p* = 0.004) (Table 3). We extracted data from this study and regrouped patients according to the intervals from the last dose of ICI to the diagnosis of COVID-19: the last dose within 90 days, the interval between 91 and 180 days, > 180 days, and without prior ICI, with mortality rates of 57%, 60%, 0%, and 25%, respectively, without significant differences between the four groups (Fig. 4).

**Table 3:** Effect of total number of previous immune checkpoint inhibitor (ICI) doses and time from last ICI dose to COVID-19 diagnosis on the development of respiratory failure due to COVID-19

	Total (*n* = 19)	Respiratory failure (*n* = 12)	No respiratory failure (*n* = 7)	*p* value
Total number of times ICI has been administered in the past, Median (min − max)	7 (1 − 32)	5.5 (1 − 18)	16 (3 − 32)	0.07^a)^
Time from last dose of ICI to COVID-19 diagnosis, Median (min − max)	144 (10 − 420)	76 (10 − 274)	221 (163 − 420)	0.004^a)*^

^*^P < 0.05 was considered statistically significant

^a)^ Mann − Whitney U test

## Discussion

This present study investigated the risk factors for respiratory failure in patients with lung cancer diagnosed with COVID-19 and treated with chemotherapy within 90 days. Our findings suggest that high CRP levels (≥ 2.75 mg/dL) and history of ICI administration within 90 days are risk factors for respiratory failure in COVID-19 patients with lung cancer. Patients with such factors may develop respiratory failure earlier than patients without these factors.

In the univariate analysis, there were no significant differences in comorbidities in the development of respiratory failure. However, male gender, smoking history, interstitial pneumonia, chronic obstructive pulmonary disease, chronic renal failure, and hypertension were associated with the respiratory failure group (Table 1). Although a multivariate analysis that included existing factors should have been performed, the small number of cases did not allow for adequate analysis. The possibility that the combined effect of the influencing factors led to the development of respiratory failure cannot be ruled out.

Regarding the association between cytotoxic anticancer drugs and the severity of COVID-19, a history of cytotoxic anticancer drug treatment within 14 days was not extracted as a risk factor for the development of respiratory failure, which contrasts with previous studies [16, 17] reporting that a history of treatment within 14 days to 1 month may influence COVID-19 severity, but is consistent with other previous studies [18, 19] reporting that cytotoxic anticancer drug treatment does not contribute to the risk of severity of COVID-19. Although there is no conclusive view on the association between cytotoxic anticancer drug therapy and COVID-19 severity, a study reporting that chemotherapy within seven days is associated with the severity of COVID-19 [20] suggests that a decrease in the neutrophil count is one factor. In this study, there was no difference in neutrophil count between the two groups, and the neutrophil counts were maintained.

CRP is a protein produced by hepatocytes in response to interleukin 6 (IL-6), and an increase in serum CRP level is a nonspecific but sensitive response to tissue injury [21]. Clinically, it is widely considered a biomarker of inflammatory and infectious diseases and directly correlates with inflammation and disease severity [22]. Therefore, an elevated serum CRP level is an important biomarker for assessing the severity of infection [23]. The results of this study is consistent with those of previous reports [3, 24] showing that high CRP levels were associated with severe disease. Although lung cancer progression has been reported to be associated with an elevated CRP level [25], the fact that 30 patients in this study had unresectable or advanced recurrent lung cancer, excluding a patient receiving adjuvant chemotherapy, and no significant differences in treatment lines were observed, suggests that the difference in CRP levels was strongly influenced by COVID-19. Therefore, an elevated CRP level may be an important risk factor for predicting COVID-19 severity, even in patients with lung cancer. Although the cutoff value of the CRP level was 2.75 mg/dL, which is similar to a previously reported value of 2.38 mg/dL [3], a large discrepancy was observed when our results were compared with the results of overseas studies [24] that reported patients with CRP > 41.8 mg/L were more severely ill; however, the median time from onset to hospitalization was 8 days [24]. In contrast, herein we speculated that the median time from the establishment of infection to positive identification was shorter because the patients were hospitalized, and SARS-CoV-2 nucleic acid detection tests were performed daily. Furthermore, the short duration between the finding of COVID-19 positivity and the blood draw (1–3 days) suggests that the blood draw may have been performed before an increase in the CRP level; therefore, the CRP level may have been low.

Ferritin and D-dimer levels exceeded the reference values in both groups, but no significant differences were found in this study. Ferritin has been reported to be elevated in patients with lung cancer [26]. Furthermore, the D-dimer level is elevated in patients with cancer [27, 28]; however, the results of the present study suggest that it may not necessarily be a risk factor for severe disease in patients with lung cancer.

Unlike patients in the non-ICI group in this study, all patients treated with ICI within 90 days had respiratory failure, suggesting its association with the severity of COVID-19. Patients with lung cancer are given various drug therapy options depending on the presence or absence of genetic mutations and the protein expression level of programmed cell death ligand 1 [29]. In recent years, cancer immunotherapy has been used to treat cancer by inhibiting the immune escape from cancer cells and has played a key role in lung cancer management [30, 31]. COVID-19 can cause acute respiratory distress syndrome in severe cases, with rapid progression of respiratory failure and a fatal course [32]. Usually, most patients with COVID-19 recover after activation of innate and acquired immunity. However, in elderly and critically ill patients, the inflammatory and immune response becomes uncontrollable, and the excessive production of inflammatory cytokines, such as IL-6, has been suggested to be involved in cytokine release syndrome, which can cause lethal pathologies [33, 34]. The immuno-stimulatory effects of ICI have also been reported to be toxic in normal cells, including lung epithelial cells, and to induce cytokine release syndrome [35, 36]. Therefore, the ICI-treated group became severely ill and suffered respiratory failure due to the massive release of cytokines.

Although the time to onset of respiratory failure was significantly faster in the high CRP group and ICI within 90 days group (Fig. 3), it should be noted that the results may be biased due to the small number of cases in this study. However, previous studies have reported that patients with cancer might have a higher risk of COVID-19 and deteriorate more rapidly than patients without cancer [7]. Our results suggest that respiratory failure may develop earlier in patients with lung cancer, especially in those with high CRP levels or a history of ICI administration. The median time to the onset of respiratory failure was 8 days in both the high CRP group and ICI within 90 days group, similar to that in a previous report [37].

Although there were no significant differences in the total number of doses of ICI or the time between the last dose of ICI and the diagnosis of COVID-19 for the development of respiratory failure, there was a trend toward higher mortality in patients who received ICI within 90 days and those who received ICI within 90 to180 days (Fig. 4). Furthermore, patients who received over three cycles of ICI have been reported to be more likely to develop severe COVID-19 [14], and the present results suggest that difference in time from the last ICI administration to the diagnosis of COVID-19 may affect the prognosis. The median time to pneumonia due to immune-related adverse events in patients receiving ICI has been reported to be 2.5 months [38], and another study reported pneumonia 9 days after the first dose [39]. If the favorable timing of pneumonia due to immune-related adverse events coincides with the time of the diagnosis of COVID-19, then more careful follow-up is necessary due to the possibility of severe disease.

The total number of ICI administrations in the group without respiratory failure was higher than that in the group with respiratory failure; although the difference was not significant, the time between the last administration and the diagnosis was longer (Table 3). It is possible that even in patients who had received numerous ICI treatments in the past, if the time between the last ICI administration and the diagnosis of COVID-19 was longer than a certain period, the effect of ICI was minimized and COVID-19 did not become more severe.

In this study, both a history of ICI administration within 90 days and high CRP level (≥ 2.75 mg/dL) were identified as risk factors. Although no significant differences were found, the incidence of respiratory failure was tended to be higher in patients with both risk factors, 100% (4/4) compared to 63.6% (7/11) in patients with only one risk factor. The risk severity of COVID-19 may be higher with two risk factors rather than with one.

Based on the results of this study, we believe that the two factors can be used as predictors of earlier and more severe disease in patients with lung cancer who also have COVID-19; therefore, these patients should be carefully monitored after disease onset.

This study had several limitations. First, it was a single-center, retrospective observational study with a limited sample size. Furthermore, a larger number of cases was required for analyses based on Kaplan–Meier curves and log-rank tests; therefore, the results of the current findings are statistically underpowered, and the numbers obtained may not be necessarily reliable. In other words, the findings of the current study should be interpreted with caution. Although studies with larger sample sizes are needed, we consider this a research limitation because of the difficulty in collecting patients with lung cancer who are also affected by COVID-19. Second, the mortality rate in Japan for people aged 60 years and older with underlying diseases affected by COVID-19 has been reported to be 12.8% [12]; however, the mortality rate in this study was high (32%), suggesting a large impact of having lung cancer as an underlying disease. However, since both comparison groups were patients with lung cancer and did not include other cancer types of cancer therefore, the effects of lung cancer did not significantly affect the quality of the results. Third, not all patients were vaccinated against SARS-CoV-2 during the investigation; therefore, the results may have differed in the context of the current widespread vaccination. However, the data in this study were obtained in a context where the vaccines and therapeutics against COVID-19 were not well developed and are purely indicative of the factors that cause severe disease due to COVID-19 in patients with lung cancer.

## Conclusions

We experienced a large cluster of cases of COVID-19 that led to the deaths of numerous patients with lung cancer in our hospital. The situation was studied, and our findings suggest that for COVID-19-affected patients undergoing chemotherapy for lung cancer, a history of ICI within 90 days and elevated CRP level (≥ 2.75 mg/dL) were found to be potential factors leading to respiratory failure. Hence, such patients require close observation and early therapeutic intervention. Our results could facilitate the development of appropriate medical care and serve as a reference for clinicians and other health care professionals to predict the severity of COVID-19 in its early stages, especially in patients with lung cancer.

## Data Availability

The datasets used and/or analyzed during the current study are available from the corresponding author upon reasonable request.

## Abbreviations

COVID-19: Coronavirus disease 2019
CRP: C-reactive protein
ICI: Immune checkpoint inhibitor
ROC: Receiver operating characteristic
SARS-CoV-2: Severe Acute Respiratory Syndrome Coronavirus
SpO_2_: Percutaneous arterial blood O_2_ saturation chain reaction
SARS-CoV-2: Severe Acute Respiratory Syndrome Coronavirus
SpO_2_: Percutaneous arterial blood O_2_ saturation
irAE: Immune-related adverse events
